# Antiproliferative Activity and *in Vivo* Toxicity of Double-Point Modified Analogs of 1,25-Dihydroxyergocalciferol

**DOI:** 10.3390/ijms161024873

**Published:** 2015-10-20

**Authors:** Justyna Trynda, Eliza Turlej, Magdalena Milczarek, Anita Pietraszek, Michał Chodyński, Andrzej Kutner, Joanna Wietrzyk

**Affiliations:** 1 Ludwik Hirszfeld Institute of Immunology and Experimental Therapy, Polish Academy of Sciences, 12 Weigla, 53-114 Wrocław, Poland; E-Mails: justyna.trynda@iitd.pan.wroc.pl (J.T.); turlej@iitd.pan.wroc.pl (E.T.); milczarek@iitd.pan.wroc.pl (M.M.); 2 Pharmaceutical Research Institute, 8 Rydygiera, 01-793 Warsaw, Poland; E-Mails: a.pietraszek@ifarm.eu (A.P.); m.chodynski@ifarm.eu (M.C.); a.kutner@ifarm.eu (A.K.); 3 Institute of Chemistry, Environmental Protection and Biotechnology, Jan Długosz University, 13/15 Armii Krajowej Ave., 42-200 Częstochowa, Poland

**Keywords:** calcitriol and analogs, 1,25-dihydroxyergocalciferol analogs, human cancer cell lines, antiproliferative activity *in vitro*, CD11b and CD14 cell-surface markers, cytoplasmic vacuolization, cell cycle, *VDR* and *MARRS* mRNA expression

## Abstract

Analogs of 1,25-dihydroxyergocalciferol, modified in the side-chain and in the A-ring, were tested for their antiproliferative activity against a series of human cancer cell lines *in vitro* and *in vivo* toxicity. The proliferation inhibition caused by the analogs was higher than that of the parent compounds, while the toxicity, measured as the serum calcium level, was lower. All analogs were able to induce, in HL-60 and MV4-11 leukemic cells, G_0_/G_1_ cell cycle arrest and differentiation expressed as morphological signs typical for monocytes. The analogs also induced the expression of CD11b and/or CD14 cell-differentiation markers. The most potent analogs, PRI-5105, PRI-5106, PRI-5201 and PRI-5202, were also able to induce vitamin D receptor (VDR) protein expression, mainly in the cytoplasmic fraction of HL-60 or MV4-11 cells. The most active analogs were the 19-*nor* ones with an extended and rigidified side-chain (PRI-5201 and PRI-5202), as in the former analogs PRI-1906 and PRI-1907. Epimerization at C-24 (PRI-5101) or introduction of an additional hydroxyl at C-23 (PRI-5104) reduced the toxicity of the analog with retained antiproliferative activity.

## 1. Introduction

Differentiation cancer therapy is defined as treatments inducing differentiation of cancer cells, and in consequence preventing further proliferation. For over 30 years, particular attention has been paid towards studies for the treatment of various cancers by means of differentiation therapy. These studies gave a strong basis for the implementation of such strategies in the clinic [1]. To date, successful differentiation therapy is achieved using several retinoids, including classical all-*trans* retinoic acid (ATRA) as well as its aromatic analogs tamibarotene and bexarotene. However, treatment with ATRA leads to frequent remission of acute promyelocytic leukemia (APL) and often results in differentiation syndrome (*i.e.*, retinoic acid syndrome) [2].

Numerous *in vitro* and *in vivo* studies, demonstrating that calcitriol (1,25-dihydroxyvitamin D_3_), an active hormonal form of vitamin D_3_, is an efficacious inhibitor of cancer cell proliferation, have supplied the justification for using this hormone in the treatment of patients suffering from leukemia and other types of cancer. It has been shown [3,4,5,6,7] that calcitriol or its analogs interact synergistically in the antitumor activity with some chemotherapeutic agents. This effect is frequently described as a result of calcitriol-induced cell differentiation [5,8,9]. Administration of potentially effective, but hyper-physiological doses of calcitriol in the treatment of cancer patients is limited by its activity regulating calcium and phosphorus metabolism and as consequence risk of hypercalcemia and hyperphosphatemia. These unsought side effects have encouraged the synthesis of new analogs, aiming to minimize calcemic activity and to increase anticancer effects [10].

In our previous studies, we tested, for their anticancer activity, both a series of vitamin D_2_ analogs with a double unsaturated side-chain and a series of vitamin D_3_ analogs with an additional one or two hydroxyls in the side-chain. We showed that the vitamin D_3_ metabolite, (24*R*)-1,24-dihydroxyvitamin D_3_ (tacalcitol, 1,24*R*-(OH)_2_D_3_, PRI-2191), demonstrated lower calcemic activity as well as lower toxicity and higher antitumor potential than calcitriol [7,11]. Our previous studies also indicated the higher antiproliferative activity of synthetic analogs of vitamin D_2_ with the extended and rigid side chain, PRI-1906 and its side-chain unsaturated homolog PRI-1907 [6,8,12,13]. PRI-1906 exhibited lower toxicity than that of calcitriol, but higher than that of PRI-2191 and antitumor activity similar to that of PRI-2191 or that of calcitriol. However, the induction of *in vivo* mammary adenocarcinoma cells differentiation by PRI-1906 [5] as well as leukemic cells *in vitro* [12] is reduced and lower than that of PRI-2191. What more, PRI-1907 is substantially more toxic than both calcitriol and PRI-1906 or PRI-2191 [5]. We have also identified a new calcipotriol derivative with diastereomeric and geometric modifications, PRI-2205, that shows a strong effect on the cell cycle, and antiproliferative activity *in vitro* [3,9,13], very low toxicity and antitumor activity *in vivo* [14,15,16].

**Figure 1:**
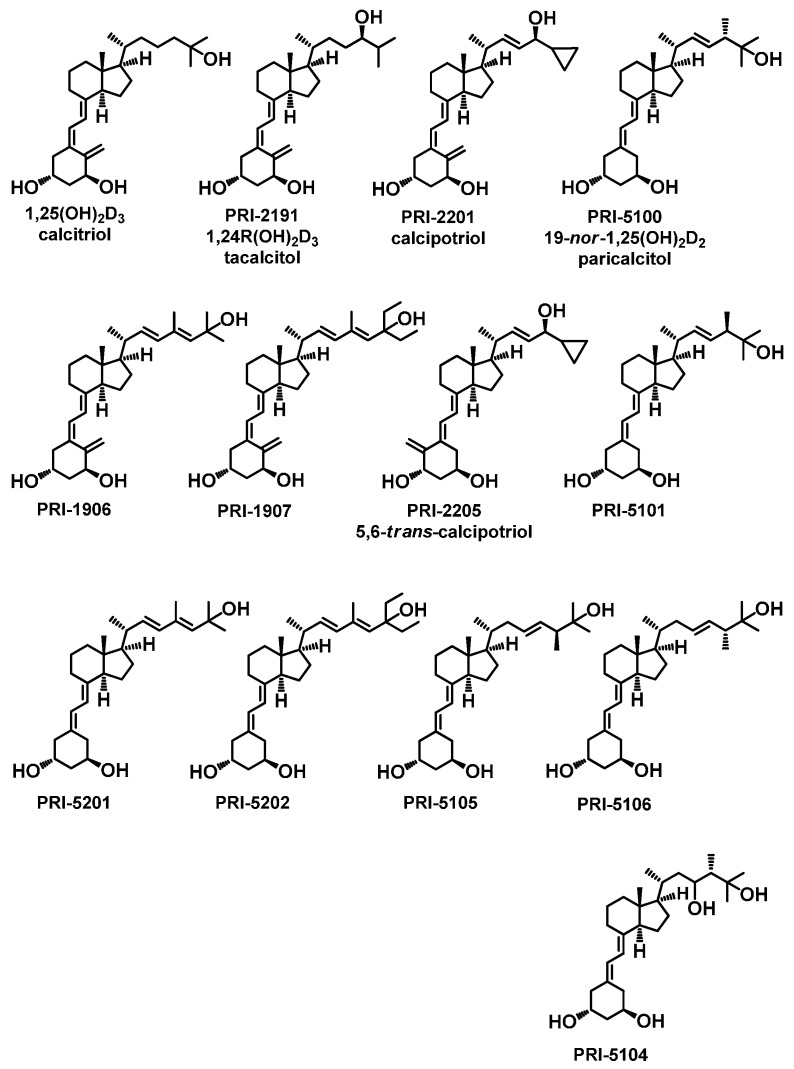
Chemical structure of analogs of 1,25-dihydroxyvitamin D.

Recently, considering the beneficial properties of previously obtained and evaluated vitamin D_2_ analogs (PRI-1906 and PRI-1907) and the 19-*nor* modification of the A-ring as in paricalcitol (PRI-5100) [17,18], we described the synthesis, as well as crystal structures of new analogs of 1,25-dihydroxyergocalciferol (PRI-5201 and PRI-5202) [19]. In this study, we showed the biological properties of these analogs, as well as those of the newly synthesized ones (Figure 1).

## 2. Results

### 2.1. Antiproliferative Activity of Analogs

The antiproliferative activity *in vitro* of all compounds were examined against human cancer cell lines. The highest antiproliferative activity of analogs was observed against three human leukemic cell lines—MV4-11, HL-60 and THP-1—in a dose-dependent manner. The IC_50_ value calculated for these cell lines were compared with those of reference compounds (calcitriol, PRI-2191, PRI-2205, PRI-1906, PRI-1907 and PRI-5100) (Table 1). PRI-5201 and PRI-5202 revealed the strongest (comparable with that of PRI-1907) proliferation inhibition of all the new analogs tested and this effect was about 10–100 times higher than that of calcitriol. When we considered the effect against individual cell lines, PRI-5105 and PRI-5106 revealed a marked activity against HL-60 and MV4-11 cells. The activity of these two analogs was comparable to that of tacalcitol and even higher than that of calcitriol. The other two analogs (PRI-5101 and PRI-5104) revealed a similar activity to that of calcitriol or calcipotriol.

**Table 1:** Antiproliferative activity *in vitro* of analogs of 1,25-dihydroxyvitamin D and that of reference analogs against HL-60, MV4-11 and THP-1 leukemia cell lines.

Compound/Analog	Cell Line/IC_50_ (nM)		
	HL-60	MV4-11	THP-1
calcitriol	44.2 ± 16.9	15.6 ± 8.7	64.6 ± 23.3
tacalcitol (PRI-2191)	8.7 ± 2.0 *	2.8 ± 0.9 *	6.4 ± 1.9
calcipotriol (PRI-2201)	21.1 ± 2.8 *	4.5 ± 0.7 *	86.3 ± 4.9
PRI-2205	277.1 ± 29.6 ^a,^*	37.5 ± 14.6 ^a,^*	151.5 ± 33.0
PRI-1906	7.5 ± 3.1 ^a,^*	3.4 ± 0.2 *	55.8 ± 4.7
PRI-1907	0.9 ± 0.4 ^a,^*	0.5 ± 0.2 ^a,^*	7.2 ± 1.2
paricalcitol (PRI-5100)	29.5 ± 9.3 ^b,c^	4.4 ± 0.9 *	48.5 ± 11.0
PRI-5101	44.9 ± 9.2 ^a,b,c^	6.3 ± 2.7 ^b,c,^*	55.3 ± 13.4 ^b,c^
PRI-5104	30.2 ± 13.3	7.2 ± 2.2 ^a,b,c^	55.3 ± 13.4
PRI-5105	12.2 ± 3.7 ^a,^*	3.0 ± 0.6 ^a,^*	33.2 ± 1.8
PRI-5106	5.1 ± 1.4 ^a,^*	0.2 ± 0.2 ^a,^*	51.1 ± 7.3
PRI-5201	0.8 ± 0.1 ^a,^*	0.9 ± 0.3 ^a,^*	1.1 ± 0.2
PRI-5202	0.5 ± 0.1 ^a,^*	0.4 ± 0.1 ^a,^*	±0.1

* compared to calcitriol; ^a^ compared to paricalcitol; ^b^ compared to tacalcitol; ^c^ compared to PRI-1907.

The remaining cell lines (Table 2) were not so sensitive to the inhibition of proliferation by the analogs. In the highest concentration used (1000 nM), the proliferation inhibition did not exceed 50%. However, PRI-5201 and PRI-5202 were the most active analogs, especially toward adherent cancer cells (MCF-7, T47D and HT-29).

**Table 2:** Proliferation inhibition of various lymphoma (Jurkat, K562, Raji), breast (MCF-7, T47D) and colon cancer (HT-29) cell lines after 120 h incubation with analogs of 1,25-dihydroxyvitamin D and referential analogs at 1000 nM concentration.

Compound/Analog	Cell Line/Proliferation Inhibition at 1000 nM Concentration					
	Jurkat	K562	Raji	MCF-7	T47D	HT-29
calcitriol	11.24 ± 7.71	30.12 ± 13.83	23.55 ± 0.44	38.4 ± 6.2	28.3 ± 17.3	39.5 ± 5.2
tacalcitol (PRI-2191)	17.72 ± 2.14	32.16 ± 10.06	27.64 ± 13.11	31.5 ± 22.8	41.8 ± 21.2	45.0 ± 10.1
calcipotriol (PRI-2201)	21.56 ± 10.55	32.37 ± 10.65	11.26 ± 2.38	28.3 ± 2.4	13.0 ± 9.0	30.6 ± 9.5
PRI-2205	15.45 ± 17.04	21.81 ± 19.51	9.90 ± 1.10	34.1 ± 9.7	34.2 ± 23.8	34.3 ± 4.3
PRI-1906	17.72 ± 2.14	32.16 ± 10.06	27.64 ± 13.11	44.5 ± 17.5	32.7 ± 22.0	30.2 ± 9.0
PRI-1907	21.60 ± 9.42	37.06 ± 9.88	27.65 ± 10.57	44.1 ± 6.3	37.6 ± 25.8	36.2 ± 8.3
paricalcitol (PRI-5100)	26.10 ± 9.18	25.41 ± 6.78	17.23 ± 0.33	37.8 ± 7.1	23.6 ± 7.7	44.4 ± 3.0
PRI-5101	9.08 ± 2.94	23.00 ± 5.84	3.99 ± 5.64	40.2 ± 12.0	13.2 ± 11.1	36.0 ± 15.4
PRI-5104	10.39 ± 5.09	33.47 ± 8.09	2.14 ± 2.85	29.3 ± 4.9	18.2 ± 14.4	29.4 ± 9.3
PRI-5105	11.64 ± 8.44	9.35 ± 3.14	2.67 ± 1.39	nt	35.2 ± 0.8	nt
PRI-5106	10.68 ± 6.72	12.22 ± 4.95	4.16 ± 3.31	nt	39.8 ± 1.5	nt
PRI-5201	35.19 ± 11.0	37.14 ± 13.11	17.41 ± 5.90	43.5 ± 10.2	52.7 ± 17.2	43.0 ± 6.4
PRI-5202	21.15 ± 16.52	30.72 ± 11.27	0	30.8 ± 6.4	37.9 ± 23.9	28.6 ± 6.7

nt—not tested.

### 2.2. Analysis of Cell Cycle Distribution of Human Leukemia Cell Lines HL-60 and MV4-11

All analogs increased the percentage of both leukemia cells in G_0_/G_1_ stage. Notwithstanding, a statistically significant accumulation of both cell lines was induced only by PRI-1907 (Table 3). In addition, a significant (*p* < 0.05) augmentation in the percentage of HL-60 cells stopped in G_0_/G_1_ phase was observed after incubation with PRI-1906, PRI-5105, PRI-5106, PRI-5201 and PRI-5202, while MV4-11 cells increased when incubated with tacalcitol (PRI-2191) and paricalcitol (PRI-5100). Almost all analogs essentially decreased the percentage of both cell lines in the S phase. No significant changes were observed in the G_2_/M cell cycle phase, with the exception of PRI-1907, where the number of MV4-11 cells was significantly decreased (Table 3).

**Table 3:** HL-60 and MV4-11 cell cycle distribution of analogs of 1,25-dihydroxyvitamin D and that of reference analogs.

Analog	HL-60			MV4-11		
	G_0_/G_1_	S	G_2_/M	G_0_/G_1_	S	G_2_/M
	Mean ± SD (%)					
control	53.4 ± 3.0	27.1 ± 0.8	17.1 ± 3.3	73.2 ± 1.8	17.9 ± 2.1	7.9 ± 0.6
EtOH	56.8 ± 2.8	26.7 ± 3.2	16.4 ± 3.7	73.1 ± 2.0	17.5 ± 1.6	7.9 ± 1.7
calcitriol	68.1 ± 6.9	13.8 ± 3.3 *	17.4 ± 2.9	78.7 ± 2.4	11.0 ±0.7 *	8.5 ± 2.6
tacalcitol (PRI-2191)	68.0 ± 6.3	13.7 ± 3.1 *	16.6 ± 5.0	82.6 ± 3.2 *	10.3 ± 2.0 *	5.8 ± 1.6
PRI-1906	70.9 ± 4.3 *	10.0 ± 5.1 *	18.3 ± 6.0	80.8 ± 3.0	9.5 ± 1.6 *	6.4 ± 0.9
PRI-1907	79.0 ± 6.0 *	8.6 ± 2.6 *	10.8 ± 4.3	83.8 ± 1.7 *	8.4 ± 1.3 *	4.7 ± 1.2 *
paricalcitol (PRI-5100)	68.9 ± 7.7	16.3 ± 5.1	14.7 ± 2.9	81.6 ± 1.8 *	9.5 ± 0.6 *	7.6 ± 1.6
PRI-5101	65.9 ± 3.2	15.7 ± 2.4 *	17.7 ± 0.9	78.3 ± 1.5	11.0 ± 2.1 *	8.8 ± 1.2
PRI-5104	66.4 ± 5.7	16.0 ± 3.1 *	16.7 ± 2.3	76.6 ± 2.5	13.2 ± 2.1	8.5 ± 1.0
PRI-5105	75.3 ± 6.3 *	11.0 ± 4.7 *	13.1 ± 1.5	80.4 ± 7.3	8.3 ± 2.9 *	7.6 ± 2.5
PRI-5106	79.2 ± 6.7 *	8.1 ± 3.6 *	11.8 ± 2.4	77.3 ± 8.9	11.4 ± 5.4	7.3 ± 1.7
PRI-5201	82.3 ± 7.1 *	7.2 ± 4.0 *	9.6 ± 3.9	80.5 ± 5.1	8.4 ± 1.9 *	7.4 ± 3.2
PRI-5202	82.3 ± 7.6 *	7.3 ± 4.0 *	9.3 ± 4.1	80.2 ± 5.2	9.6 ± 2.1 *	7.0 ± 2.1

The results are presented as a mean (%; ±SD) percentage of the cell population qualified to one of the three groups: cells in phase G_0_/G_1_, S and G_2_/M. The percentage of death cells did not exceed 5%. * Indicates statistically significant (*p* ≤ 0.05) (tested compounds (1 nM) *vs.* ethanol: EtOH 0.001%). Statistical analysis: Mann-Whitney *U* Test.

### 2.3. CD11b and CD14 Expression on HL-60 and MV4-11 Cells Incubated with Analogs for 120 h

**Figure 2:**
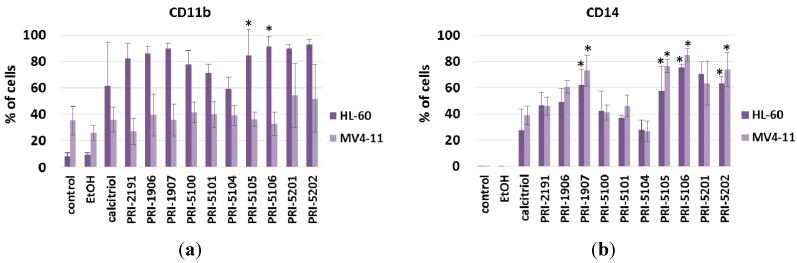
Expression of CD11b (**a**) and CD14 (**b**) surface markers on HL-60 and MV4-11 cells exposed to analogs of 1,25-dihydroxyvitamin D and reference analogs (1 nM) for 120 h. Results of four experiments are presented as the percentage of positive cells (% ± SD). Control cells *vs.* ethanol (EtOH; 0.001%)—not significant. All treatment groups differed significantly from the EtOH control; the exception are results for CD11b on MV4-11—not significant. Results marked with asterisks differed significantly (*p* ≤ 0.05) from calcitriol. Statistical analysis: Mann-Whitney *U* Test.

HL-60 and MV4-11 cells were incubated with either calcitriol or analogs at 1 nM concentration for 120 h. Control, untreated cells possessed a very low expression of CD11b and CD14 molecules. After exposure to all tested compounds, the expression these characteristic for monocytes cell-surface markers, essentially increased (with the exception of CD11b on MV4-11 cells). A statistically significant increase, as compared to control or ethanol treated cells, was observed for two analogs, PRI-5105 and PRI-5106, in the case of CD11b antigen on HL-60 cells. Moreover, the same two analogs, as well as PRI-1907 and PRI-5202, increased the percentage of HL-60 and MV4-11 cells expressing CD14 antigen (*p* > 0.05). The expression of CD14 and CD11b after incubation with the analogs confirmed their impact on cell differentiation. In cells treated with paricalcitol (PRI-5100), PRI-5101 and PRI-5104, the elevation in monocytes-specific surface markers was rather weak and not statistically significant (Figure 2).

### 2.4. Cytoplasmic Vacuolization of Leukemic Cells after Incubation with Vitamin D Analogs

**Figure 3:**
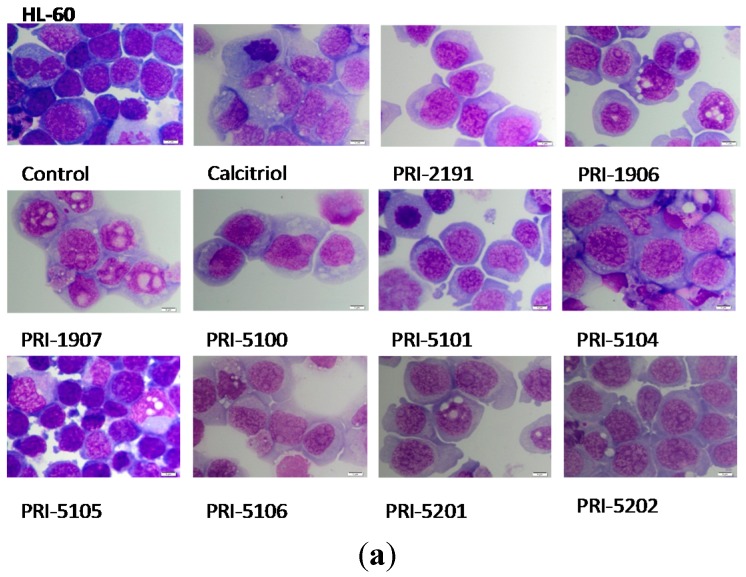

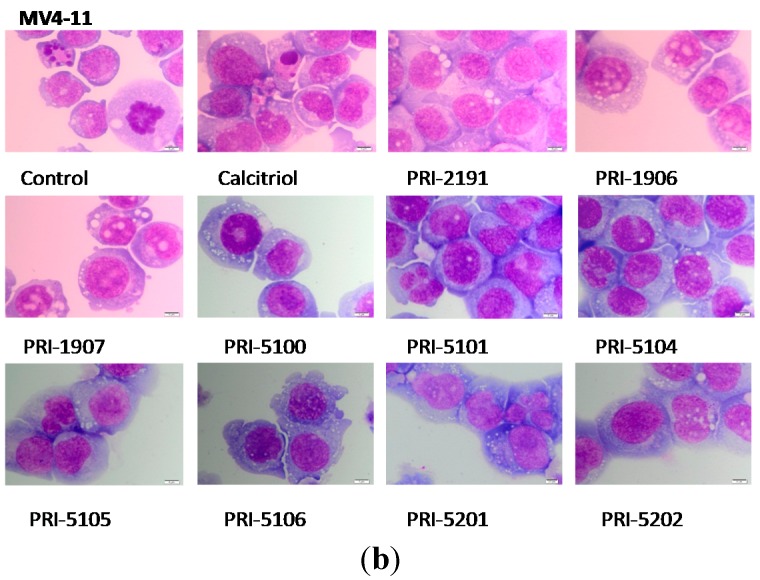
Cytoplasmic vacuolization induced in leukemic cells by vitamin D analogs. Human HL-60 (**a**) or MV4-11 (**b**) leukemia cells were cultured for 72 h in the presence or absence of vitamin D analogs (1 nM) and subjected to Giemsa staining. Representative results are shown. Scale bar = 5 μm.

After treatment of HL-60 or MV4-11 cells with vitamin D analogs at 1 nM concentration, the cells lost their regular shape, part of them acquired amoeboid shapes, cytoplasm lost its basophilic character, and the ratio of nucleus: cytoplasm changed in favor of cytoplasm. Nuclei became irregular and vacuoles could be observed in the cytoplasm. In general, cells acquired a more mature phenotype and monocyte-like appearance (Figure 3).

### 2.5. VDR and MARRS mRNA Expression

**Figure 4:**
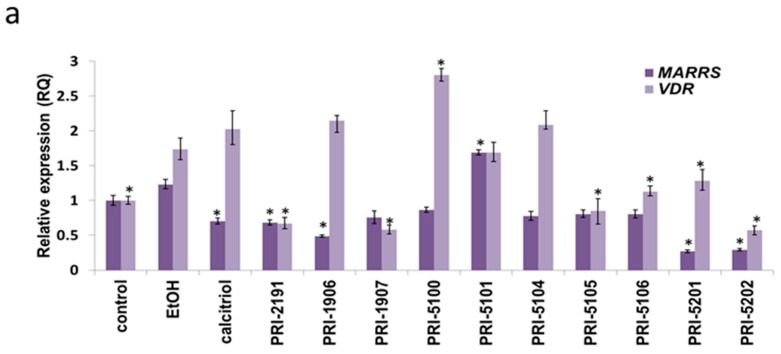

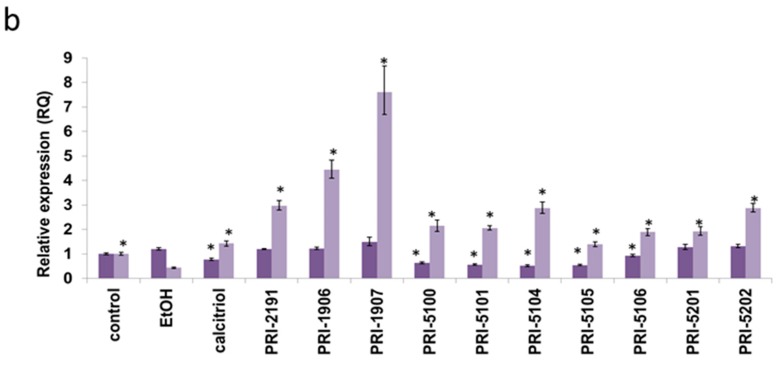
The impact of analogs of 1,25-dihydroxyvitamin D on *MARRS* and *VDR* mRNA expression in HL-60 (**a**) and MV4-11 (**b**). HL-60 and MV-4-11 cells were exposed to 1 nM of calcitriol or its analogs for 72 h. *MARRS* and *VDR* were normalized relative to *GAPDH* in each well, and each sample value for the gene expression was then normalized to the calibrator value (control sample was chosen as calibrator = 1). Three independent RNA extractions were performed for each cell line and the final result of gene expression was calculated for at least 2 repetitions. Data analysis was performed with the use of Data Assist version 3.1 (Freeware by Applied Biosystems, Carlsbad, CA, USA). * *p* < 0.05 as compared to EtOH (0.001%) control.

The influence of all analogs on *VDR* and *MARRS* mRNA expression was analyzed. The non-treated cells were arbitrarily used as a control in calculations. Ethanol control (EtOH) itself influenced the expression of mRNA, especially *VDR* mRNA, in both cell lines. A statistically significant increase in *VDR* mRNA in HL-60 cells treated with PRI-5100 and the decrease in cells treated with PRI-2191, PRI-1907, PRI-5105, PRI-5106, PRI-5201 and PRI-5202 (Figure 4a) was observed. In MV4-11 cells, the changes of *VDR* mRNA level significantly increased after treatment with all compounds, as compared to EtOH. However, PRI-1906 and PRI-1907 were the strongest inducers of *VDR* mRNA increasing its expression 4.4 to 7.6 times, respectively (Figure 4b).

The changes in *MARRS* mRNA levels were not as high as those observed for *VDR*. A significant increase in *MARRS* mRNA was observed in HL-60 cells incubated only with PRI-5101. The other vitamin D analogs decreased the expression of *MARRS* mRNA, as compared to EtOH. A statistically significant decrease, following incubation with calcitriol, PRI-2191, PRI-1906, PRI-5201 and PRI-5202, was also noticed in HL-60 cells (Figure 3a). Decreased expression of *MARRS* mRNA was also observed in MV4-11 cells treated with calcitriol, PRI-5100, PRI-5101, PRI-5104 and PRI-5105 (*p* < 0.05). However, PRI-5201 and PRI-5202, the analogs with the highest potential to decrease the *MARRS* mRNA in HL-60 cells (~0.3, *p* < 0.05, Figure 3a), only slightly increased the level of this protein in MV4-11 cells (~1.3-fold, Figure 4b).

### 2.6. VDR Protein Expression

**Figure 5:**
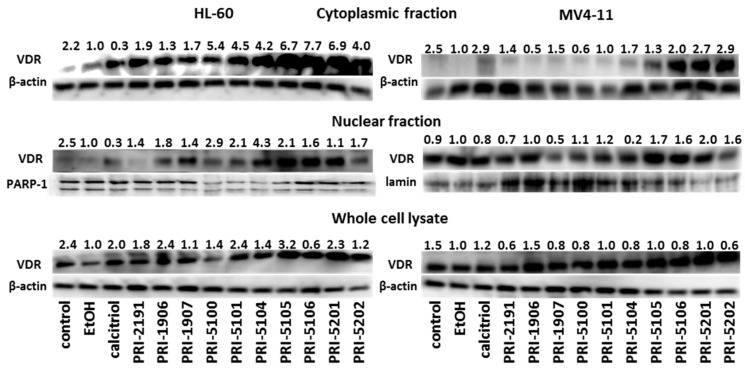
HL-60 or MV4-11 cells incubated with analogs of 1,25-dihydroxyvitamin D up-regulate VDR expression in the cytoplasmic cell fraction. Representative Western blots of VDR and appropriate loading control expression in MV4-11 cells incubated in the presence of the 1 nM of vitamin D analogs for 72 h. Relative VDR protein expression was determined by the density of the VDR bands from Western blots of cells incubated in the presence of reference compounds and analogs at the indicated concentrations (1 nM). The density of the VDR bands was normalized to the density of the loading controls and then to the VDR bands of cells incubated with the ethanol control (EtOH, 0.001%). One out of two independent experiments is presented.

The HL-60 cells incubated with vitamin D analogs (with exception of PRI-5106) showed higher VDR protein levels in whole cell lysates as compared to the ethanol control, but these were similar to those of non-treated cells. In nuclear and especially in cytoplasmic fractions, paricalcitol (PRI-5100) as well as all new analogs showed a tendency to increase VDR protein levels (Figure 5).

The MV4-11 whole cell lysates, independently of incubation mode, showed similar VDR protein levels. However, this protein expression increased in the cytoplasmic fraction, especially after incubation with calcitriol, PRI-5104, PRI-5106, PRI-5201 and PRI-5202. In the nuclear fraction of these cells, only a slight (~2-fold) increase in VDR expression was noticed after incubation with PRI-5105, PRI-5106, PRI-5201 and PRI-5202 (Figure 5).

### 2.7. Preliminary Toxicity Studies of New Analogs in Vivo

Following our previous observations [5,7], we used the analogs at a dose of 10 μg/kg/day administered to mice for two consecutive days. There was a 10% body weight decrease only for analog PRI-5202 by the third day of observation, when the animals were sacrificed (Table 4). A statistically significant increase in calcium levels was observed in serum of mice treated with calcitriol, tacalcitol (PRI-2191) and PRI-1907. The mice treated with PRI-5101, PRI-5104 and PRI-5105 showed the lowest calcium level, similar to control animals. In the remaining groups, an increase in calcium level was observed. However, these values were not statistically significant (Table 4). Glucose serum level have tendency to increase in mice treated with PRI-5105 and PRI-5202, however not in a significant manner. Similarly, the biochemical parameters describing liver and kidney functions do not differ significantly between groups of mice. The exception is decreased level of aspartate aminotransferase (ASP) in mice treated with PRI-5201 and urea in mice treated with PRI-5101 (Table 4).

**Table 4:** Selected serum biochemical parameters and body weight change in mice injected subcutaneously with analogs of 1,25-dihydroxyvitamin D and those of reference analogs.

Group	*n*	Ca (mg/dL)	P (mg/dL)	ASP (U/L)	ALT (U/L)	Urea (mM/L)	CRE (μM/L)	Glucose (mM/L)	Body Weight Change (%)
control	5	10.35 ± 0.21	7.74 ± 0.64	37.62 ± 18.01	99.64 ± 37.69	7.33 ± 0.65	17.72 ± 4.10	7.78 ± 1.05	2.28
calcitriol	6	14.44 ± 1.43 *	8.21 ± 0.49	31.54 ± 7.28	102.46 ± 21.75	9.02 ± 1.08	20.64 ± 4.19	6.74 ± 1.22	2.08
tacalcitol (PRI-2191)	5	14.09 ± 0.55 *	7.84 ± 1.02	31.68 ± 4.08	92.8 ± 14.95	7.91 ± 0.74	18.30 ± 3.80	9.18 ± 0.92	1.64
PRI-1906	5	11.94 ± 0.51	9.12 ± 1.52	28.16 ± 2.95	91.44 ± 11.57	6.61 ± 0.52	16.82 ± 3.10	7.52 ± 1.37	0.00
PRI-1907	5	17.61 ± 1.20 *	7.24 ± 0.26	31.54 ± 4.56	102.76 ± 11.57	7.83 ± 1.23	17.70 ± 1.76	6.53 ± 0.96	−2.96
paricalcitol (PRI-5100)	5	12.87 ± 0.21	8.40 ± 0.36	35.68 ± 3.24	106.85 ± 22.12	7.42 ± 0.69	17.52 ± 2.64	7.52 ± 1.27	0.92
PRI-5101	5	10.62 ± 0.68 **	8.07 ± 0.52	33.25 ± 3.30	86.10 ± 9.36	6.80 ± 0.66 *	18.40 ± 1.76	8.43 ± 1.80	0.29
PRI-5104	5	10.62 ± 0.78 **	7.32 ± 0.49	29.3 ± 4.03	103.96 ± 8.76	5.54 ± 2.24	15.80 ± 1.38	8.03 ± 0.67	1.58
PRI-5105	3	10.98 ± 0.25 **	7.84 ± 0.09	32.03 ± 2.09	76.87 ± 8.52	7.60 ± 0.36	12.30 ± 1.22	10.69 ± 1.67	−1.82
PRI-5106	3	12.61 ± 0.08	7.69 ± 0.52	32.30 ± 1.82	86.23 ± 19.97	7.42 ± 0.34	12.53 ± 0.95	7.95 ± 0.55	2.70
PRI-5201	5	12.76 ± 1.04	8.25 ± 1.44	31.33 ± 3.44 *	95.78 ± 7.64	7.35 ± 0.99	17.10 ± 2.17	7.81 ± 0.94	0.00
PRI-5202	5	13.50 ± 1.20	7.63 ± 0.48	27.23 ± 3.12	90.33 ± 2.16	10.66 ± 0.44	22.16 ± 2.01	10.91 ± 0.86	−10.26

* *p* < 0.05 as compared to control; ** *p* < 0.05 as compared to PRI-1907; Statistical analysis: Kruskal-Wallis multiple comparison test; Body weight change (%) calculated on day 3; initial body weight/body weight on day 3 × 100; Calcitriol or its analogs or 80% propylene glycol were administered subcutaneously at the dose of 10 μg/kg once a day, for two consecutive days; On day 3, blood was collected. *n*—number of mice; ASP—aspartate aminotransferase; ALT—alanine aminotransferase; CRE—creatinine.

**Figure 6:**
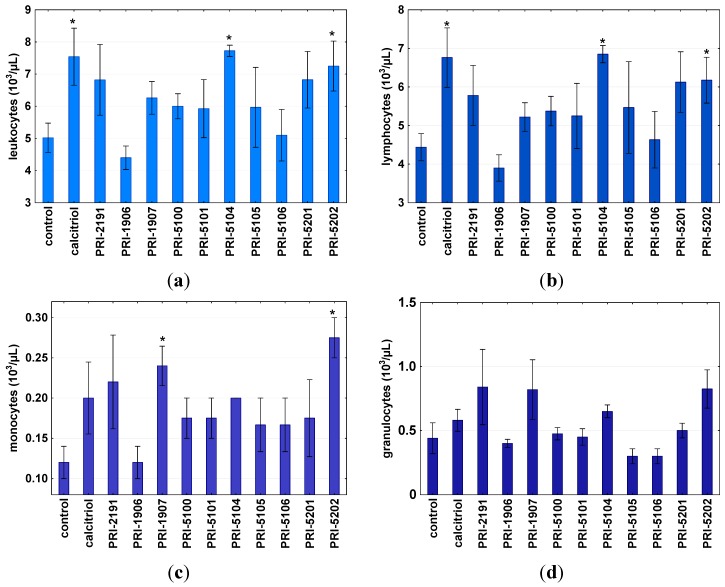
Leukocytes (**a**) as well as lymphocytes (**b**), monocytes (**c**) and granulocytes (**d**) counted in mice treated with the analogs. Calcitriol or its analogs or 80% propylene glycol were administered subcutaneously at the dose of 10 μg/kg once a day, for two consecutive days. On day 3, blood was collected. * Indicates statistically significant (*p* ≤ 0.05) (tested compounds *vs.* control mice treated with 80% propylene glycol-control). Statistical analysis: Mann-Whitney *U* Test. Results are presented as mean ± standard deviation (SD).

Blood morphology parameters were also analyzed. After such a short-term treatment, we observed a statistically significant increase in white blood cells in mice treated with calcitriol, PRI-5104 and PRI-5202 (Figure 6a). This was a result of an increased number of lymphocytes (Figure 6b). However, an increased number of monocytes (Figure 6c) was also observed after treatment with calcitriol, PRI-2191 and, in a significant manner, with PRI-1907 and PRI-5202, as the most toxic analogs. The changes in the number of blood granulocytes were not statistically significant (Figure 6d).

**Figure 7:**
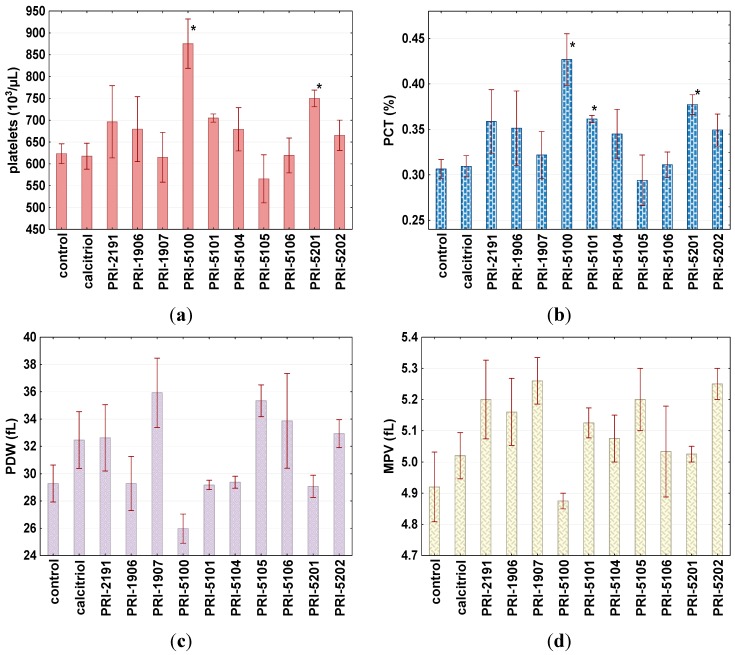
Platelet numbers and other platelet parameter distributions in mice treated with the tested compounds. Calcitriol or its analogs or 80% propylene glycol were administered subcutaneously at the dose of 10 μg/kg once a day, for two consecutive days. On day 3, blood was collected. (**a**) Platelet number; (**b**) plateletcrit (PCT); (**c**) platelet distribution width (PDW); (**d**) mean platelet volume (MPV). * Indicates statistically significant (*p* ≤ 0.05) (tested compounds *vs.* control mice treated with 80% propylene glycol-control). Statistical analysis: Mann-Whitney *U* Test. Results are presented as mean ± standard deviation (SD).

An increase in the number of platelets was also observed (Figure 7a) in mice treated with PRI-5100 and PRI-5201 (*p* < 0.05). This was correlated with a significant increase in plateletcrit (PCT, Figure 7b) and the tendency to decrease platelet distribution width (PDV, Figure 7c) and mean platelet volume (MPV, Figure 7d) in the case of paricalcitol (PRI-5100). Besides PRI-5100, MPV showed a tendency to increase in all treated animals (Figure 7d).

**Figure 8:**
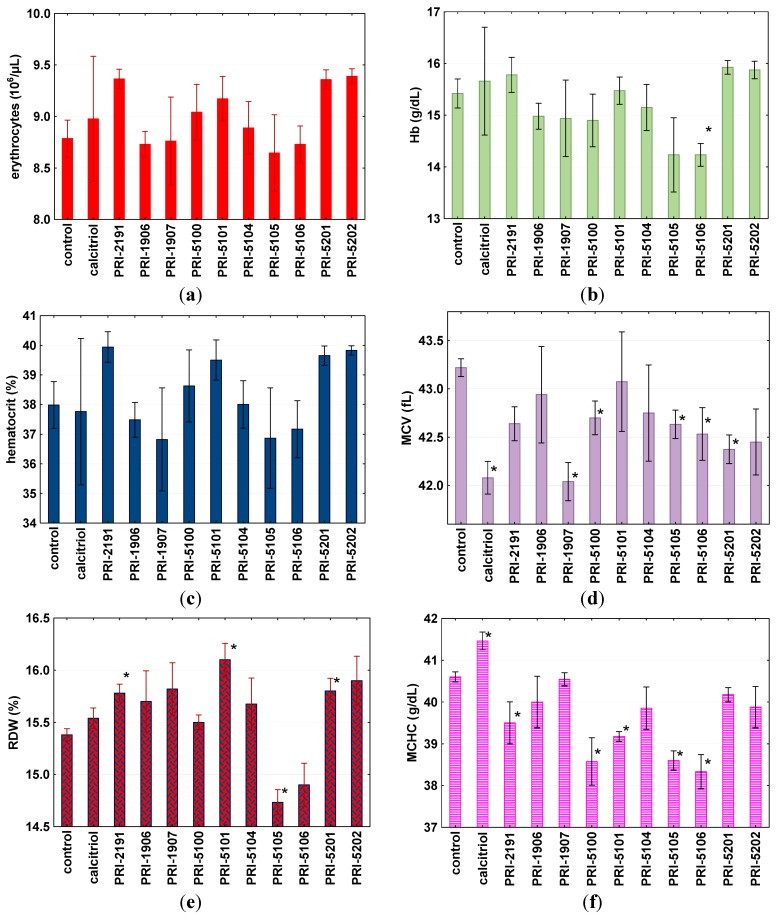

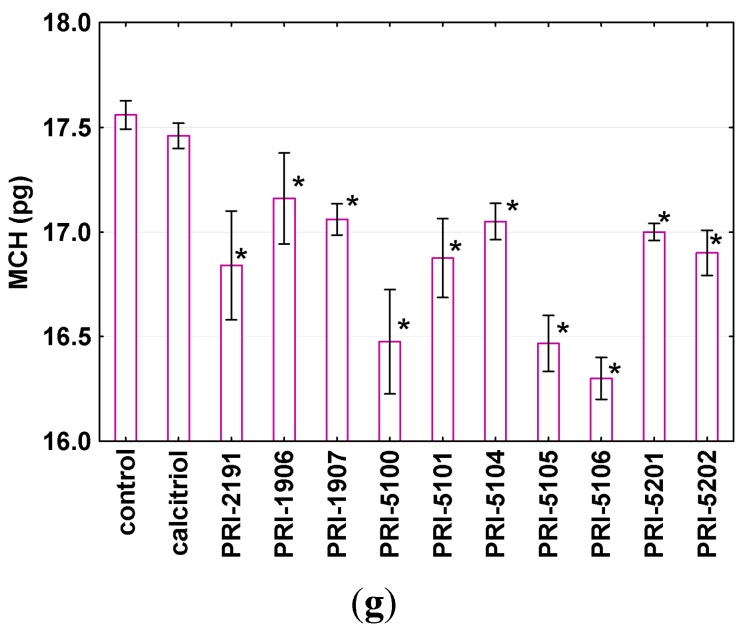
Erythrocyte numbers (**a**) and other erythrocyte parameters (**b**–**g**) in mice treated with the tested compounds. * Indicates statistically significant (*p* ≤ 0.05) (tested compounds *vs.* control mice treated with 80% propylene glycol). Statistical analysis: Mann-Whitney *U* Test. Results are presented as mean ± standard deviation (SD). Calcitriol or its analogs or 80% propylene glycol were administered subcutaneously at the dose of 10 μg/kg once a day, for two consecutive days. On day 3, blood was collected. (**a**) Erythrocytes number; (**b**) hemoglobin (Hb); (**c**) hematocrit; (**d**) mean cell volume (MCV); (**e**) red distribution width (RDW); (**f**) mean corpuscular hemoglobin concentration (MCHC); (**g**) mean corpuscular hemoglobin (MCH).

Neither the number of erythrocytes nor hematocrit changed significantly; however, the highest values of both parameters were observed in mice treated with PRI-2191, PRI-5101, PRI-5201 and PRI-5202. On the other hand, the lowest values of hemoglobin, red distribution width (RDW), mean corpuscular hemoglobin (MCH) and mean corpuscular hemoglobin concentration (MCHC) were observed in mice treated with PRI-5105 and PRI-5106. In general, such parameters as mean cell volume (MCV) and MCH were decreased (Figure 8).

## 3. Discussion

Numerous studies have been performed to find the most effective vitamin D analog or active metabolite in anticancer treatment. However, the majority of analogs used to date are single-point modified ones and have largely been reviewed already [20,21]. Here, we analyzed the biological properties of double-point modified analogs of 1,25-dihydroxyergocalciferol and compared them with reference analogs bearing both modifications separately [19].

The three most active new analogs (PRI-5106, PRI-5202 and PRI-5201) induced relatively high serum calcium levels. However, these values were significantly lower than those of PRI-1907—the most active, but also the most toxic analog [5]. What more, antiproliferative activity of these new analogs is comparable to that of PRI-1907. Less active compounds, e.g., tacalcitol, paricalcitol or calcitriol, increased calcium levels in a similar manner to the three most active new analogs. Therefore, we concluded that 19-*nor* modification of the A-ring, combined with a mono-homologated vitamin D_2_-like side-chain and branched side chains at C-25, as in PRI-1907—resulted in PRI-5202 with the retained activity, but significantly reduced toxicity. However, 19-*nor* modification of PRI-1906, present in the other most active analog PRI-5201, increased not only its activity, but also its toxicity, as compared to both parent compounds: paricalcitol (PRI-5100) or PRI-1906.

The (24*R*) diastereomer of paricalcitol, PRI-5101, did not significantly differ from its parent compound in its inhibition of cell proliferation or induction of differentiation. However, its toxicity, measured as the induction of calcium in serum, was significantly lower than that of paricalcitol (PRI-5100) (*p* < 0.05). Moreover, this compound did not alter the pattern of platelets as had been observed for paricalcitol (Figure 7). Interestingly, the activity of analog PRI-5101 and one of its side-chain homologs, PRI-5106, differ significantly. The antiproliferative and pro-differentiation activity of PRI-5106 is almost 10-fold higher than that for PRI-5101, but its toxicity is only slightly increased. Analogs PRI-1906 and PRI-5105, despite average antiproliferative activity, showed the lowest toxicity (serum calcium level); comparable to that of PRI-5101 and PRI-5104. In contrast to PRI-1906, which had rather low pro-differentiation potential (Figure 2 and [5,22]), PRI-5105 increased the percentage of cells showing CD11b or CD14 monocyte differentiation markers, similar to highly active PRI-1907. Vitamin D analogs are known inducers of the myeloid leukemia cells differentiation with subsequent antiproliferative activity [12,22,23]. Paricalcitol also induced monocytic differentiation of HL-60 cells, as estimated by the elevated expression of CD14 cell surface marker and cell morphological changes [24]. Our studies confirmed the pro-differentiating activity of known analogs (on HL-60, as well as MV4-11 cell lines) and showed that new analogs also increased CD14 and/or CD11b differentiation markers as well as induced morphologic signs of monocytic differentiation such as oval, irregular, or serrated nuclei and plentiful cytoplasm with vacuoles.

There is now increasing evidence that the mechanisms of action of vitamin D compounds are dependent on nuclear receptors (nVDR), as well as on the specific binding sites, named Membrane Associated, Rapid Response Steroid (MARRS) binding proteins [25]. These two distinctive proteins showed distinguished affinities for vitamin D analogs which may be helpful in some clinical conditions. For example, in leukemia patients, it would be favorable to promote the activities of the nVDR for malignant cells differentiation or the intestinal MARRS stimulation would be desirable in the elderly [25,26,27]. Wu *et al.* showed that translocation of MARRS and NFκB to the nucleus, upon co-stimulation with calcitriol and phorbol ester, preceded the expression of the monocyte/macrophage phenotype of NB4 promyelocytic leukemia and suggested a role for *MARRS* as a regulator of gene expression in the nucleus [27]. Moreover, the involvement of MARRS protein in calcitriol activity in breast cancer has been described [28]. Therefore, we decided to analyze the influence of a panel of new analogs on the expression of VDR and MARRS, in terms of both mRNA and protein (in the case of VDR) levels, toward two leukemia cell lines. The most active new analogs (PRI-5105, PRI-5106 and PRI-5201, PRI-5202) intensively increased the expression of VDR proteins (this was not observed on the mRNA level) mainly in cytoplasmic cell fractions. This might suggest that these analogs are weak VDR degradation activators [29].

The biological activity of new analogs of 1,25-dihydroxyergocalciferol was presented. All new analogs revealed high antiproliferative activity, mainly toward leukemic cells, which was correlated with blocked cell cycle in G_0_/G_1_ phase and cell monocytic differentiation. The toxicity of the analogs, measured as serum calcium level, was lower than in parent compounds. Moreover, four of new analogs, PRI-5105, PRI-5106 and PRI-5201, PRI-5202, were able to significantly increase the level of VDR proteins in cytoplasmic cell fractions.

## 4. Experimental Section

### 4.1. Cells

Human leukemia HL-60, MV-4-11, K562, THP-1 and lymphoma Jurkat, Raji cells (all from American Type Culture Collection, Rockville, MD, USA) were cultured in RPMI 1640 medium (Gibco, Scotland, UK) with 10% fetal bovine serum, 4.5 g/L glucose, 2 mM l-glutamine, 1.5 g/L sodium bicarbonate and 1.0 mM sodium pyruvate (all from Sigma-Aldrich Chemie GmbH, Steinheim, Germany). RPMI 1640 + Opti-MEM (1:1) (both from Gibco) with 5% fetal bovine serum (Thermo-Fisher Scientific Oy, Vataa, Finland) and 2 mM l-glutamine were used to culture T47D human breast cancer cells (ATCC, Rockville, MD, USA) and HT-29 human colon cancer cells (German Cancer Research Center, DKFZ, Heidelberg, Germany). The HT-29 culture media included 1.0 mM of sodium pyruvate (Sigma-Aldrich Chemie GmbH). MCF-7 human breast cancer cells (ATCC) were cultured in Eagle medium (IIET, Wroclaw, Poland) supplemented with 10% fetal bovine serum (all from Sigma-Aldrich Chemie GmbH), 2 mM l-glutamine and 1% MEM non-essential amino acid solution (Sigma-Aldrich Chemie GmbH). All these cell lines are maintained at the Ludwik Hirszfeld Institute of Immunology and Experimental Therapy, Wroclaw, Poland.

The T47D and MCF-7 culture media were supplemented with 0.8 mg/L of insulin (Sigma-Aldrich Chemie GmbH). All media used in experiments were supplemented with 100 μg/mL streptomycin and 100 units/mL penicillin (both from Polfa Tarchomin S.A., Warsaw, Poland). All cells were passaged before reaching confluence and grown at 37 °C with a 5% CO_2_ humidified atmosphere.

### 4.2. Compounds

The vitamin D compounds were tested: calcitriol, PRI-2191, PRI-2205, PRI-1906, PRI-1907, PRI-5100, PRI-5101, PRI-5104, PRI-5105, PRI-5106, PRI-5201 and PRI-5202 (Figure 1). The compounds were synthesized at the Pharmaceutical Research Institute, Warsaw, Poland. All compounds were prepared in absolute ethanol to the concentration of 10^−4^ M. For *in vitro* examination, analogues were soluted in culture medium to reach the requisite concentrations (ranging from 1 to 1000 nM). All tested compounds were dissolved in 99.8% ethanol, then diluted in 80% propylene glycol to reach the required concentrations and administered subcutaneously (*s.c*.) to mice (50 μL/10 g of body weight). Exposure to light in all procedures was minimized to prevent degradation of the light-sensitive reagents.

### 4.3. Antibodies

Fluorescein isothiocyanate (FITC)-conjugated CD11b monoclonal antibodies (moAb) (clone ICRF44) and phycoerythrin (PE)-conjugated CD14 moAb (clone UCHM-1) were purchased from Sigma Aldrich (Sigma-Aldrich, St. Louis, MO, USA). Isotype control FITC-conjugated and PE-conjugated IgG1 immunoglobulins from murine melanoma were also purchased from Sigma-Aldrich. All antibodies were used at concentrations recommended by the suppliers.

### 4.4. Anti-Proliferative Assay in Vitro

The leukemic, MCF-7, T47D (1 × 10^4^ cells per well), and HT-29 (2 × 10^3^ cells per well) cells were seeded in 96-well plates (Sarstedt, Numbrecht, Germany) 24 h before application of compounds. The assay was performed after exposure to varying concentrations of the analog for 120 h. For adherent cells; a sulforodamine B (SRB) assay was performed; with an MTT (3-(4,5-dimethylthiazol-2-yl)-2,5-diphenyl tetrazolium bromide) assay for leukemia cells; as described previously [9]. The results as an inhibitory concentration 50—the dose of the tested compounds which inhibits proliferation of 50% of the cell population were calculated. IC_50_ values for each individual experiment were estimated and mean values ± SD are presented in the tables [30]. The analogs in each concentration was tested in triplicate in a single experiment, which was made in more than 3 repeats.

### 4.5. MV4-11 and HL-60 Cell Incubation for Cell Cycle Analysis, Differentiation Assay, Immunocytochemistry and Western Blot

The HL-60 and MV4-11 human leukemia cells were plated (2 × 10^5^ cells/mL of culture medium) on 24-well plates (Sarstedt, Numbrecht, Germany). The cells were incubated with the analogs (1 nM) for 120 h (flow cytometric analyses) or 72 h (cytochemistry, real time PCR, Western blot). Ethanol (used to dissolve), in the appropriate dilutions connected with highest concentration used for the analogs (0.01%), was used as a control. After incubation, the cells were collected, washed in phosphate-buffered saline (PBS) and counted in a hemacytometer.

#### 4.5.1. Cell Cycle Analysis

Cells (1 × 10^6^) were 2 times washed in cold PBS and then were fixed in 70% ethanol at −20 °C for 24 h. After washing in PBS, the cells were treated using RNAse (50 μg/mL, Fermentas, St. Leon-Rot, Germany) at 37 °C for 1 h. After that, the cells were treated with propidium iodide (50 μg/mL, Sigma-Aldrich Chemie GmbH) at 4 °C for 30 min and the content of the cellular DNA was read out using BD LSR Fortessa II (Becton Dickinson, San Jose, CA, USA). The percentage of stained cells was determined by ModFit LT version 3.2 developed by Verity Software House, USA and Flowing Software version 2.5.1 (freeware) developed by Perttu Terho, Turkey (version from 4.11.2013).

#### 4.5.2. MV4-11 and HL-60 Differentiation Assay

Expression assessment of CD11b and CD14 were made by flow cytometry method, 5 × 10^5^ HL-60 cells in 100 μL of PBS (with 2% fetal bovine serum) were mixed with monoclonal antibody in the proper volume. Incubation of the cells were made by using ice bath for 45 min and after that two times washed in 2 mL of PBS. As a negative control FITC and PE-conjugated IgG1 were used. The expression of the cell surface antigens was assessed by BD LSR Fortessa II (Becton Dickinson, San Jose, CA, USA). Data analysis was determined using Flowing Software version 2.5.1.

#### 4.5.3. Lysate Preparation and Western Blot

In order to prepare cytoplasmic and nuclear fractions, 5 × 10^6^ cells/sample were removed from 24-well plates. The cells were rinsed using PBS and then the cytoplasmic buffer (NP40 0.5%, NaCl 100 mM, TRIS 20 mM) with protease and phosphatase inhibitors (Sigma-Aldrich, Poznan, Poland) was added. Cells were kept on ice for 15 min and then centrifuged (5 min, 358× *g*, 4 °C). The supernatant was moved to pure tube (cytosolic fraction) and the residue was washed with cytoplasmic buffer two times and then treated with RIPA with protease and phosphatase inhibitors (all from Sigma-Aldrich, Poznan, Poland). After 10 min incubation, all probes were frozen at −80 °C. Before protein determination, samples used as a nuclear fraction were centrifuged (10,000× *g*, 10 min, 4 °C). The whole cell lysates were prepared using RIPA only, which contained the phosphatase and protease inhibitors. The protein concentration was determined with the use of protein assay (DC Protein Assay; Bio-Rad Laboratories, Hercules, CA, USA). The protein (20 μg for the sample) was separated in a gradient ready polyacrylamide gel (15-wells Mini-PROTEAN, Biorad, Warsaw, Poland) and then transferred to a polyvinylidene difluoride membrane (PVDF, 0.45 μm; GE Healthcare, Amersham, Little Chalfont, UK). Then, the membranes (4 °C) in 1% blocking reagent (Membrane Blocking Agent; GE Healthcare, Amersham) prepared in PBS overnight were blocked. Next day, the membranes were rinsed three times (×10 min) with the use of 0.05% PBS/Tween-20 (PBST) and incubated for one hour at room temperature with a primary antibody: rabbit anti-VDR, anti-lamin, anti-PARP-1 and anti-ERp57 antibodies (all from Santa Cruz Biotechnology Inc., Santa Cruz, CA, USA) or rabbit anti-β-actin (Sigma-Aldrich, Poznan, Poland). Then, membranes were rinsed three times (×10 min) with the use of 0.1% PBST and incubated for 60 min with the secondary anti-rabbit antibody conjugated with alkaline phosphatase (GE Healthcare, Amersham). The membranes were finally rinsed three times with the use of 0.1% PBST and incubated for 30 min with a ECF substrate to obtain the fluorescence product (ECF, GE Healthcare, Amersham). Fluorescence was detected using the Carestream Image Station 4000MM PRO (Carestream Health, Woodbridge, CT, USA). Densitometric analysis of the membranes with the use of ImageJ 1.46r (National Institutes of Health, Bethesda, MA, USA) were carried out.

#### 4.5.4. Cytospin Staining

A total of 100 μL of cells (0.5 × 10^6^/mL) were added to a slide chamber (SuperFrost, Menzel-Glasser, Braunschweig, Germany) and then centrifuged at 400 rpm (18× *g*) for 10 min at room temperature (Shandon Cytospin, Thermo Scientific, Waltham, MA, USA). After centrifugation, the slides were allowed to dry in the air for 24 h. The samples were fixed in 100% ice-cold methanol for 20 min and stained with May-Grunwald dye (Merck-Millipore, Darmstadt, Germany) diluted 1:1 in PBS (pH 7.6) for 5 min. After water rinsing, the specimens were stained with Giemsa dye (Merck-Millipore) diluted 1:9 in PBS (pH 7.6) for 15 min. After rinsing with water, the slides were allowed to dry. Almost all procedure was made at room temperature, with a small exception noted above.

A microscope with a 100× oil immersion or 20× normal objective was used to take photographs (Olympus model CX41, Olympus Europa Holding GMBH, Hamburg, Germany).

### 4.6. Real Time PCR

To examine *MARRS* and *VDR* mRNA expression, HL-60 and MV-4-11 cells were cultured in 24-well plates (2 × 10^5^ cells per mL). Then, the cells were treated with 1 nM calcitriol or its analogs for 72 h. Total RNA was isolated using TRIzol Reagent (Invitrogen, Carlsbad, CA, USA) according to the manufacturer’s protocol RNA quantity and purity was estimated spectrophotometrically at 260 nm using NanoDrop 2000 (Thermo Fisher Scientific, Waltham, MA, USA) and the quality of RNA was evaluated by agarose electrophoresis. To obtain total cDNA, a 1 μL of enzyme of high-capacity cDNA archive iScript kit (BioRad, Hercules, CA, USA) was applied to reverse transcribe 1 μg of total RNA. Real time PCR was performed with Viia 7 (Applied Biosystems by Life Technologies, Carlsbad, CA, USA). The specific oligonucleotide primer pairs, labeled with a reporter fluorescent dye (FAM) at the 5′ end, and the specific FAM TaqMan probe used for *MARRS* (*PDIA3* assay ID: Hs00607126_m1) and *VDR* (assay ID: Hs01045840_m1) and a predesigned *GAPDH* assay used as an endogenous control were obtained from Life Technologies. Real Time PCR reactions were carried out in a total volume of 10 μL including TaqMan Universal PCR Master Mix, FAM TaqMan probe, forward primer, reverse primer, and TaqMan Universal PCR Master Mix (Applied Biosystems, Foster, CA, USA). The following protocol were used: 95 °C for 10 min, followed by 40 cycles of two step PCR, including denaturation at 95 °C for 15 s and annealing/extension at 60 °C for 1 min. The amplification efficiency of *MARRS* and *VDR* were normalized using the endogenous control, *GAPDH*. The gene expression for each sample value was normalized relative to the calibrator value (control sample was selected as calibrator = 1). The target genes relative quantification were evaluated by using comparative Ct method. Three independent RNA isolations were conducted for each cell line and the final result of gene expression was calculated for at least 2 repetitions. Data were analyzed by using Data Assist v. 3.1 (freeware by Applied Biosystems).

### 4.7. Animal Experiments

C57BL6/J/cmdb female (Medical University of Bialystok; Bialystok, Poland) were maintained in specific pathogen-free (SPF) conditions. All *in vivo* experiments according to *EU Directive 2010/63/EU* were performed and by the 1st Local Committee for Experiments with the Use of Laboratory Animals were approved, Wroclaw, Poland (project identification code: 28/2013, date of approval: 17 July 2013).

#### Calcemic Activity, Hepatic and Kidney Function, Blood Morphology

Calcitriol, its analogs or 80% propylene glycol were administered subcutaneously (10 μg/kg once a day) for two days. On the third day, blood was collected. The blood morphology and serum calcium, phosphate, alanine transaminase, aspartate transaminase, urea, creatinine and glucose levels were evaluated in each blood sample using the analyzer Mythic 18 (C2 Diagnostics, Montpellier, France) and Cobas c 111 z ISE (Roche Diagnostics Ltd., Rotkreuz, Switzerland), respectively. The number of mice per group: 3–6 (Table 4).

### 4.8. Statistical Analysis

Data were analyzed by the Mann-Whitney *U* test and one-way ANOVA followed by Tukey HSD test. Differences were considered significant at *p* less than 0.05.

## 5. Conclusions

New analogs of 1,25-dihydroxyergocalciferol showed high antiproliferative activity. The most active were the 19-*nor* analogs with extended and rigidified side-chains (PRI-5201 and PRI-5202), as in the former analogs PRI-1906 and PRI-1907. The toxicity of analogs PRI-5201 and PRI-5202 was lower than that of the parent compounds. Moreover, epimerization at C-24 (PRI-5101) or introducing additional hydroxyl at C-23 (PRI-5104) diminished the toxicity of the analog with the retained antiproliferative activity. The analogs blocked cell cycle in the G_0_/G_1_ phase and induced cell differentiation as well as increased VDR level in cytoplasmic fractions of HL-60 or MV4-11 leukemia cell lysates.
